# Vasorin Deletion in C57BL/6J Mice Induces Hepatocyte Autophagy through Glycogen-Mediated mTOR Regulation

**DOI:** 10.3390/nu14173600

**Published:** 2022-08-31

**Authors:** Lichao Yang, Xiaojing Cheng, Wei Shi, Hui Li, Qi Zhang, Shiping Huang, Xuejing Huang, Sha Wen, Ji Gan, Zhouxiang Liao, Junming Sun, Jinning Liang, Yiqiang Ouyang, Min He

**Affiliations:** 1 School of Public Health, Guangxi Medical University, Nanning 530021, China; 2 Laboratory Animal Center, Guangxi Medical University, Nanning 530021, China; 3 Life Sciences Institute, Guangxi Medical University, Nanning 530021, China; 4 Key Laboratory of High-Incidence-Tumor Prevention & Treatment, Guangxi Medical University, Ministry of Education, Nanning 530021, China

**Keywords:** vasorin (*Vasn*), autophagy, glycogen, mTOR, ULK1

## Abstract

Abnormal vasorin (*Vasn*) expression occurs in multiple diseases, particularly liver cancers. *Vasn* knockout (KO) in mice causes malnutrition, a shortened life span, and decreased physiological functions. However, the causes and underlying mechanisms remain unknown. Here, we established *Vasn* KO C57BL/6J mice by using the CRISPR/Cas9 system. The animals were weighed, and histology, immunohistochemistry, electronic microscopy, and liver function tests were used to examine any change in the livers. Autophagy markers were detected by Western blotting. MicroRNA (miRNA) sequencing was performed on liver samples and analyses to study the signaling pathway altered by *Vasn* KO. Significant reductions in mice body and liver weight, accompanied by abnormal liver function, liver injury, and reduced glycogen accumulation in hepatocytes, were observed in the *Vasn* KO mice. The deficiency of *Vasn* also significantly increased the number of autophagosomes and the expression of LC3A/B-II/I but decreased SQSTM1/p62 levels in hepatocytes, suggesting aberrant activation of autophagy. *Vasn* deficiency inhibited glycogen-mediated mammalian target of rapamycin (mTOR) phosphorylation and activated Unc-51-like kinase 1 (ULK1) signaling, suggesting that *Vasn* deletion upregulates hepatocyte autophagy through the mTOR-ULK1 signaling pathway as a possible cause of diminished life span and health. Our results indicate that *Vasn* is required for the homeostasis of liver glycogen metabolism upstream of hepatocyte autophagy, suggesting research values for regulating *Vasn* in pathways related to liver physiology and functions. Overall, this study provides new insight into the role of *Vasn* in liver functionality.

## 1. Introduction

Vasorin (*Vasn*), also known as Slit-like 2 (Slitl2) or anti-TNFα-induced apoptosis (ATIA), is a highly conserved glycoprotein of 673 amino acids encoded by the *Vasn* gene, which acts like an anti-apoptotic factor that plays a role in protecting cells from death against TNF- or hypoxia-induced apoptosis [1]. *Vasn* is a transmembrane glycoprotein highly expressed during early embryonic development in mice [2]. In adult mice, the expression of *Vasn* is more abundant in the coronary artery, aorta, kidney, lung, liver, testis, and ovarian follicles [3,4]. Abnormal expression of *Vasn* occurs in multiple vascular diseases [5] and cancers [6,7,8,9,10]. Even so, to date, there is no direct evidence pointing to the association of human diseases or phenotypes with variations in the *Vasn* gene [2]. Increasing studies have added to our understanding of the close relationship between *Vasn* and liver disease [6,11,12]. *Vasn* has been found to promote the proliferation and migration of hepatoma cells and inhibit apoptosis in vitro, and the involvement of *Vasn* abnormality in liver cancers has been investigated [6]. Since autophagy is activated as an adaptive response and plays a crucial role in regulating liver physiology and in balancing liver metabolism under stressful conditions [13], it is reasonable to suspect that there may be a direct link between *Vasn* regulation and hepatocyte autophagy progress in order to maintain liver homeostasis. However, whether and how vasorin contributes to hepatocyte autophagy remain unknown.

A recent study accidentally found that *Vasn* knockout (KO) mice could not survive more than 30 days in the same conditions as wild-type (WT) mice, due to unknown reasons [2,4]. Coincidentally, a similar phenomenon was also found in one of our latest studies, where significant body weight reduction and poor health status by cardiac hypertrophy were observed in *Vasn* KO mice in comparison to normal mice [14]. Hence, we hypothesize the possible presence of malnutrition or metabolic problems aside from the known cardiac issue. Since the liver is the largest solid digestive organ that plays a pivotal role in multiple physiological processes (digestion, nutrient processing and storage, energy metabolism, protein synthesis, detoxification, etc.) [15], and malnutrition has been associated with liver disease [16], these adverse conditions in *Vasn* KO mice may be potentially related to some negative changes in the liver. Metabolism of glycogen and lipids is tightly regulated in healthy hepatocytes under normal physiological conditions [17,18]. The inability of the liver to properly manipulate and maintain the balance of glycogen and lipid metabolism are obvious manifestations of impaired regulation in situations of weight loss and malnutrition, as in liver injury and other liver diseases [16,19,20]. Autophagy, as a cellular process mobilizing intracellular nutrient resources, plays an important role in contributing to survival during unfavorable growth conditions, maintaining liver homeostasis in normal physiological conditions, and in the response to metabolic dysregulation [21,22]. However, no evidence has yet revealed the direct link between *Vasn* expression and hepatocyte autophagy. Considering the global need to understand the impact of autophagy on liver physiology and functions, this study, for the first time, examined liver histology and cellular autophagy associated with the deletion of *Vasn* in C57BL/6J mice, following a series of tests that relate pathway discovery and metabolic alterations to explore the underlying mechanism. There has been no report on the deficiency of *Vasn* leading to liver pathology until now. Our results demonstrate that *Vasn* deficiency induces hepatocyte autophagy and thereby liver injury accompanied by liver glycogen reduction in mice through a possible mechanism in the regulation of the glycogen-mediated mTOR-ULK1 signaling pathway.

## 2. Materials and Methods

### 2.1. Animals

The animal study was approved by the Institutional Animal Ethical Committee of Guangxi Medical University (approval no. 201809086). The methods were carried out in accordance with the approved guidelines. Animals used for *Vasn* KO in the study were of the C57BL/6J background. Mice were fed a chow diet (research diet), housed in a temperature-controlled room under a 12 h light/12 h dark cycle, and in specific pathogen-free (SPF) conditions. Heterozygote *Vasn* KO mice, *Vasn*^+/−^, and homozygote *Vasn* KO mice, *Vasn*^−/−^, were generated by the Shanghai Model Organisms Center, Inc. Four guide RNAs were designed based on the upstream and downstream sequences of *Vasn* exons2. *Vasn* gRNA and Cas9 mRNA prepared by in vitro transcription were injected into zygotes. F0 male mice were identified by PCR with genomic DNA in tail tissue as a template, using two primer pairs, P1/P2 and P3/P4. Positive F0 male mice were mated with C57BL/6 J female mice to breed F1 mice. F2 mice were obtained by F1 mice mated with F1 mice. Genotypes of F1 and F2 mice were determined by PCR from purified mouse tail DNA. Suckling mice were numbered by clipping the fingers, and their genotype was determined 2 weeks after birth. Mice were euthanized at 3 weeks, as the *Vasn* KO mice cannot survive more than 30 days. Since suckling mice (half of the males and females) were used for this study and placed in the same cage as their mother, they only received nutrition from their mother’s breast milk, unlike the juveniles and adult mice given food pellets and purified water.

### 2.2. Liver Function Tests (LFTs)

Serum was obtained for biochemical analysis by centrifuging the whole blood from mice at 1500× *g* for 10 min. Globulin (GLB) was determined by the OLYMPUS AU2700 automatic biochemical analyzer (OLYMPUS, Tokyo, Japan). The BECKMAN COULTER Chemistry Analyzer AU5800 (BECKMAN COULTER, Brea, CA, US) was used to measure the serum liver function index, including albumin (ALB), total protein (TP), alkaline phosphatase (ALP), alanine aminotransferase (ALT), total bilirubin (TBIL), direct bilirubin (DBIL), indirect bilirubin (IBIL), cholinesterase (CHE), and γ-glutamyl transferase (GGT).

### 2.3. miRNA Sequencing

Total RNA was isolated from the livers of *Vasn*^+/−^, *Vasn*^−/−^, and WT mice. The quantity and quality of RNA were determined by a Fragment Analyzer (Agilent Technologies, Santa Clara, CA, USA), and samples with an RNA integrity number (RIN) above 7.0 and 28S/18S above 1.5 were considered acceptable. The isolated RNAs were used for preparing small RNA libraries and then sequenced on the BGISEQ-500 platform (BGI). After data filtering, the remaining tags were called “clean tags”. Bowtie2 [23] was used to map clean reads to the reference genome and to other sRNA databases. The small RNA expression level was calculated as transcripts per kilobase million (TPM) [24]. Correction for false positive (type I errors) and false negative (type II) errors was performed using the False Discovery Rate (FDR) method [25]. In the analysis, differentially expressed miRNAs were defaulted with FDR ≤ 0.001 and multiples of more than 2-fold.

Multiple types of software were used to seek accurate targets. Generally, RNAhybrid [26], miRanda [27], or TargetScan [28] were used to predict targets. Hierarchical clustering was performed for differentially expressed miRNAs. The Kyoto Encyclopedia of Genes and Genomes (KEGG) [29] was used to outline the pathway enrichment analysis.

### 2.4. Transmission Electron Microscopy (TEM)

Mice were sacrificed and the liver was immediately isolated, cut with blades to no more than 1 mm^3^ volume, and fixed in 3% glutaraldehyde for more than 2 h. After washing with 0.1 mol/L PBS three times, samples were post-fixed in 1% osmium tetroxide for 1–2 h. Ultrathin sections were cut and stained with uranyl acetate and lead citrate. Finally, the samples were analyzed with a TEM (Hitachi H7650, Tokyo, Japan).

### 2.5. Histopathology

Mice were sacrificed, and the liver was immediately isolated, fixed in 4% paraformaldehyde for 24 h, and embedded with paraffin. The embedded specimens were sectioned to a thickness of 4 μm and stained with hematoxylin and eosin (H&E).

For immunohistochemistry analysis, after dewaxing and hydration, antigen retrieval was mediated by high pressure in a citrate buffer (pH 6.0). Sections were blocked with 10% normal goat serum for 30 min at room temperature (RT). After incubation with endogenous peroxidase inhibitor for 30 min at RT and washing thrice with PBS, the primary antibody was incubated at 37 °C for an hour. The primary antibodies and the dilutions used are as follows: SQSTM1/p62 Rabbit mAb (Cell Signaling Technology, Danvers, MA, USA; #23214, 1:250), LC3A/B Rabbit mAb (Cell Signaling Technology, Danvers, MA, USA; #12741, 1:100), Phospho-ULK1 (Ser555) Antibody (Affinity Biosciences, OH, USA; #AF7148,1:100), and Anti-mTOR (phosphor S2448) antibody (Abcam, Cambridge, UK; ab109268, 1:100). The primary antibody was detected by a biotinylated secondary antibody and visualized using an HRP-conjugated SP system. Slides were washed and stained with DAB substrate solution and hematoxylin. After dehydration, transparency, and sealing, slides were photographed with the EVOS FL Auto Cell Imaging System (Thermo Fisher Scientific, Waltham, MA, USA). IHC quantification was performed using Image-Pro Plus v6.0 software (Media Cybernetics, Silver Spring, MD, USA).

### 2.6. Periodic Acid Schiff (PAS) Glycogen Staining

PAS glycogen staining was performed using the Glycogen Periodic Acid Schiff (PAS/Hematoxylin) Stain Kit (Solarbio, Beijing, China), according to the manufacturer’s protocol. Slides were photographed with the EVOS FL Auto Cell Imaging System (Thermo Fisher Scientific, Waltham, MA, USA), and PAS quantification was performed using Image-Pro Plus software.

### 2.7. Quantitative Analysis and Detection of Glycogen

Quantitative analysis of glycogen was performed using a Glycogen Content Assay Kit (Solarbio, Beijing, China). In brief, 0.1~0.3 g of liver tissue was put into a 10 mL centrifuge tube and 0.75 mL of extract was added, followed by boiling for 20 min (covered tightly to prevent water loss), with shaking once every 5 min to fully mix. After the tissue was completely dissolved, we took out the centrifuge tube for cooling, fixed the volume to 5 mL with distilled water, mixed well, centrifuged at 8000× *g* at 25 °C for 10 min, and took the supernatant to measure the absorbance at 620 nm. The calculation was performed as follows:

Glycogen (mg/mg prot) = (C_standard_ × V1) × (A3 − A1) ÷ (A2 − A1) ÷ (V1 × Cpr) ÷ 1.11 = 0.09 × (A3 − A1) ÷ (A2 − A1) ÷ Cpr

### 2.8. Quantitative Real-Time Reverse-Transcription PCR (qRT-PCR)

Twelve mice from wild-type, *Vasn*^+/−^, and *Vasn*^−/−^ groups were then allocated to different blocks (N1, N2, N3, and N4) using a randomized block design. Total RNA was isolated using the TransZol Up Plus RNA Kit (TransGen Biotech, Beijing, China). The RNA concentration was determined by a Biospec-nano spectrophotometer (Shimadzu, Tokyo, Japan). Total RNA was transcribed into complementary DNA by an miRNA First Strand cDNA Synthesis Kit (Tailing Reaction) (Sangon, Shanghai, China). The U6 was used as a loading control. The primers Universal PCR Primer R and Universal U6 Primer F were provided in the kit. Other sequences of primers are shown in Table S1. qRT-PCR was performed using the ChamQ Universal SYBR qPCR Master Mix on a LightCycler 480 Instrument II rapid high-throughput plate-based real-time PCR amplification and detection instrument (Roche). Relative quantifying analysis was performed with the 2^−ΔΔCT^ method.

### 2.9. Western Blot Assay

Mice liver tissues were cut into tiny pieces and added with RIPA lysis buffer containing protease inhibitor. The mixture was ground thoroughly with a tissue grinder until it was fully cracked. After centrifugation at 12,000× *g* for 5 min, the total protein in supernatant was obtained for subsequent protein detection. The concentration of protein was determined using a BCA Protein Assay Kit (Beyotime, Shanghai, China). Then, 60 μg protein samples were separated in 12% SDS-PAGE gel and transferred onto PVDF (polyvinylidene difluoride) membrane (Millipore, Bedford, MA, USA). After blocking, the membrane was incubated with appropriate primary antibodies at 4 °C overnight. The primary antibodies were as follows: SQSTM1/p62 Rabbit mAb (Cell Signaling Technology, #23214, 1:1000), LC3A/B Rabbit mAb (Cell Signaling Technology, #12741, 1:1000), Phospho-ULK1 (Ser555) Antibody (Affinity Biosciences, #AF7148,1:1000), ULK1 Antibody (Affinity Biosciences, #DF7588, 1:1000), mTOR Rabbit mAb (Cell Signaling Technology, 2983S, 1:1000), Anti-mTOR (phosphor S2448) antibody (Abcam, ab109268, 1:1000), and GAPDH Rabbit mAb (Cell Signaling Technology, #5174, 1:3000). After washing with TBST, the membrane was incubated with anti-rabbit IgG HRP-linked antibody (Cell Signaling Technology, #7074, 1:5000), and protein bands were visualized by chemiluminescence (Thermo Fisher Scientific, Waltham, MA, USA) using an iBright FL1000 imaging system (Thermo Fisher Scientific, Waltham, MA, USA). The protein expression levels were quantified using ImageJ v1.48 software (NIH, Bethesda, MD, USA).

### 2.10. Statistical Analyses

The data were analyzed by GraphPad Prism 7.0 (Graph Pad Software, La Jolla, CA, USA). Two-tailed Student’s *t* test was used to evaluate the significance of the difference between two groups of data in the experiments. One-way analysis of variance (ANOVA) was used to determine the statistically significant difference among three or more different groups of data in the experiments. *p* ≤ 0.05 was considered to be statistically significant.

## 3. Results

### 3.1. Vasn Deficiency Causes Liver/Body Weight Loss

**Figure 1:**
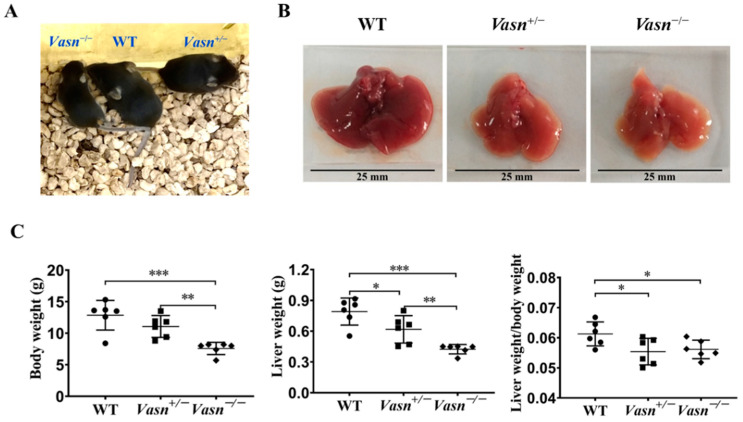
Liver weight/body weight loss in *Vasn*^−/−^ and *Vasn*^+/−^. (**A**) Photos of *Vasn*^−/−^, *Vasn*^+/−^, and WT mice. (**B**) Photos of livers separated from *Vasn*^−/−^, *Vasn*^+/−^, and WT mice. (**C**) Liver weight/body weight loss in *Vasn*^−/−^ and *Vasn*^+/−^ mice. * *p* < 0.05, ** *p* < 0.01, *** *p* < 0.001. Data are represented as mean ± SD of six mice per group.

*Vasn* plays an important role in liver disease and cellular pathways [6,11,12]. Hence, we generated *Vasn* KO mice using CRISPR/Cas9 in the laboratory, as described, to investigate the connection between *Vasn* expression and liver physiology/histology. The construction strategy and genotype of newborn mice are shown in Figure S1A. The electrophoresis results for PCR products showed that a 562 bp DNA fragment containing the sequence of *Vasn* was amplified in the genomic DNA of WT (*Vasn*^+/+^) C57BL/6 and *Vasn*^+/−^ mice, but did not appear in that of the *Vasn*^−/−^ mice (Figure S1B), suggesting the successful and complete knockout of *Vasn*. The *Vasn* knockout in C57BL/6 mice was further verified by DNA sequencing of the PCR products and DNA sequence alignment. The sequencing results revealed that the 2384 bp DNA fragment containing the *Vasn* sequence was successfully deleted from the genome of C57BL/6 in *Vasn*^−/−^ mice (Figure S1C). Afterwards, during the breastfeeding, the overall sizes of the *Vasn*^−/−^ individuals were smaller than any of the WT or the *Vasn*^+/−^ mice (Figure 1A). All mice were sacrificed, weighed, dissected, and sampled at 3 weeks of age. Unlike the normal liver features of WT mice, we intuitively noticed that the livers separated from *Vasn*^+/−^ mice were significantly smaller, with thinner margins and lighter or even pale pigmentation; these features became more pronounced in most livers of *Vasn*^−/−^ mice (Figure 1B). Significant reductions in not only body weight but also liver weight and the liver-to-body weight ratio were detected in *Vasn*^−/−^ mice compared to *Vasn*^+/−^ mice or WT (Figure 1C). It is likely that the deficiency of *Vasn* caused poor growth and anemia in the liver. Nearly all the *Vasn*^−/−^ mice had significant weight loss problems of both the body and liver, suggesting that the deletion of *Vasn* may cause nutritional deprivation or possible growth retardation/dysplasia in young mice.

### 3.2. Vasn Deficiency Leads to Pathological Damage in the Liver

**Figure 2:**
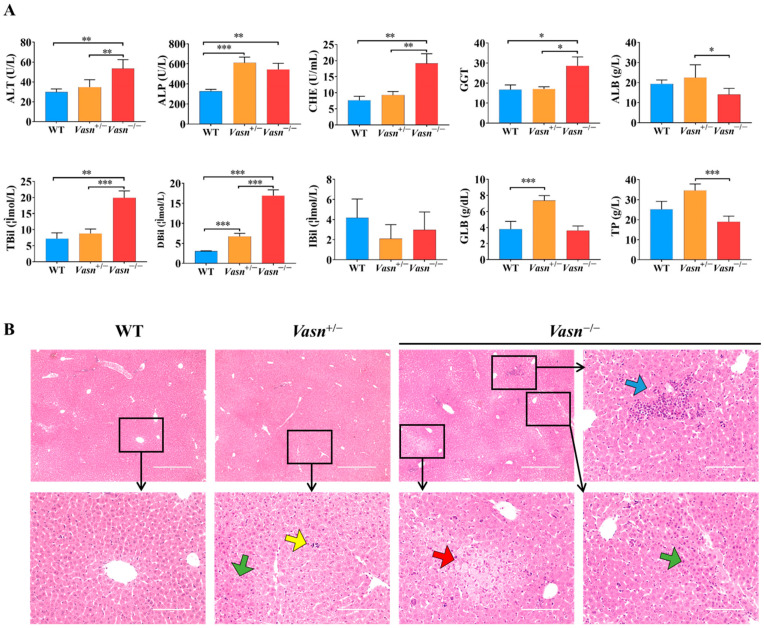
Liver injury induced in *Vasn*^−/−^ and *Vasn*^+/−^. (**A**) Serum liver function tests suggest that liver injury was induced in *Vasn*^−/−^ and *Vasn*^+/−^ mice. * *p* < 0.05, ** *p* < 0.01, *** *p* < 0.001. (**B**) H&E staining of liver from *Vasn*^−/−^, *Vasn*^+/−^, and WT mice at 3 weeks of age; yellow arrows show spotty necrosis, green arrows show inflammation, blue arrows show slice necrosis, and red arrows show ballooning degeneration. Data are expressed as mean ± SD of three independent experiments.

To further explore the reason for the reduction in liver weight, serum biochemistry tests for liver function and histopathological analysis were performed. As shown in Figure 2A, significantly high levels of ALP, ALT, TBIL, DBIL, CHE, and GGT in serum were observed in *Vasn*^−/−^ mice compared to the WT mice. Moreover, the serum levels of ALP, ALT, GLB, and DBIL were elevated in *Vasn*^+/−^ mice compared to the WT mice. The statistical comparison of biochemical indices indicates abnormal liver function in *Vasn* KO mice, where the severity of liver dysfunction seems to be directly related to the degree of complete knockout of *Vasn*. Furthermore, hepatic histopathology was examined based on H&E staining on the mouse liver sample sections. The histological examination of the liver tissues of *Vasn*^−/−^ mice revealed obvious degeneration, including spotty necrosis (yellow arrows), patchy necrosis (blue arrows), acidophilic degeneration (green arrows), and vacuolization (red arrows) (Figure 2B). In the case of *Vasn*^+/−^ mice, only few instances of spotty necrosis, inflammatory cells, and alleviated liver damage were observed. We can clearly see that there is a progressive severity of liver histopathology from WT to *Vasn*^+/−^ to *Vasn*^−/−^, which is in line with the basic hypothesis of liver pathology caused by *Vasn* knockout in this study.

### 3.3. Vasn Deficiency Leads to Glycogen Depletion in the Liver

**Figure 3:**
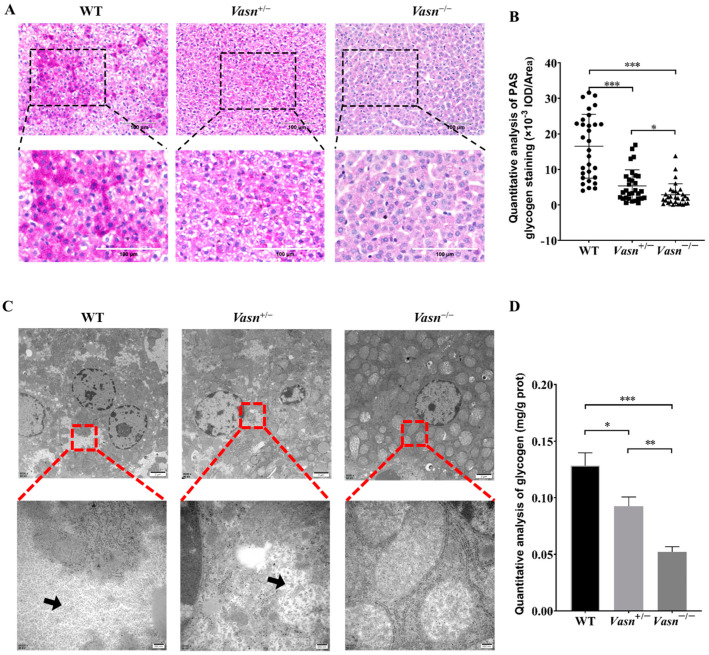
The glycogen content decreased significantly in *Vasn*^−/−^ and *Vasn*^+/−^ mice. (**A**) Periodic acid Schiff (PAS) staining detected the presence of glycogen in liver tissues of *Vasn*^−/−^, *Vasn*^+/−^, and WT mice at 3 weeks of age. (**B**) Quantitative IOD analysis of the PAS staining area. (**C**) TEM observation of glycogen in liver tissues of *Vasn*^−/−^, *Vasn*^+/−^, and WT mice at 3 weeks of age; black arrows show glycogen particles. (**D**) Quantitative analysis of the glycogen content; data are expressed as mean ± SD of three independent experiments. * *p* < 0.05, ** *p* < 0.01, *** *p* < 0.001.

According to the unexpected observation of abnormal morphology of hepatocytes (especially the ballooning and dystrophy-like features), we hypothesize that there might be a problem with cellular energy storage in the liver of *Vasn* KO mice. Thus, we detected the glycogen content in the liver tissue of the mice. Through PAS staining, we observed much smaller glycogen staining areas in the *Vasn*^+/−^ and *Vasn*^−/−^ groups compared to the WT, especially with only a small amount of glycogen appearing in the *Vasn*^−/−^ group (Figure 3A,B). The loss of glycogen was also supported by the observation of cellular ultrastructure using a transmission electron microscope (TEM) [30]. As shown in Figure 3C, there were many glycogen granules in the form of “rosettes” in the cytoplasm of wild-type hepatocytes, while fewer glycogen granules were in *Vasn*^+/−^ and were almost absent in the cytoplasm of *Vasn*^−/−^ hepatocytes. Quantitative analysis of glycogen by the anthrone method also confirmed that the deletion of *Vasn* led to the reduction in glycogen, which was consistent with the results of morphological observation (Figure 3D). Collectively, these results are indicative of glycogen reduction in *Vasn* KO mice, which might be an important mechanism of hepatocyte autophagy induction.

### 3.4. Vasn Deficiency Induces Hepatocyte Autophagy

**Figure 4:**
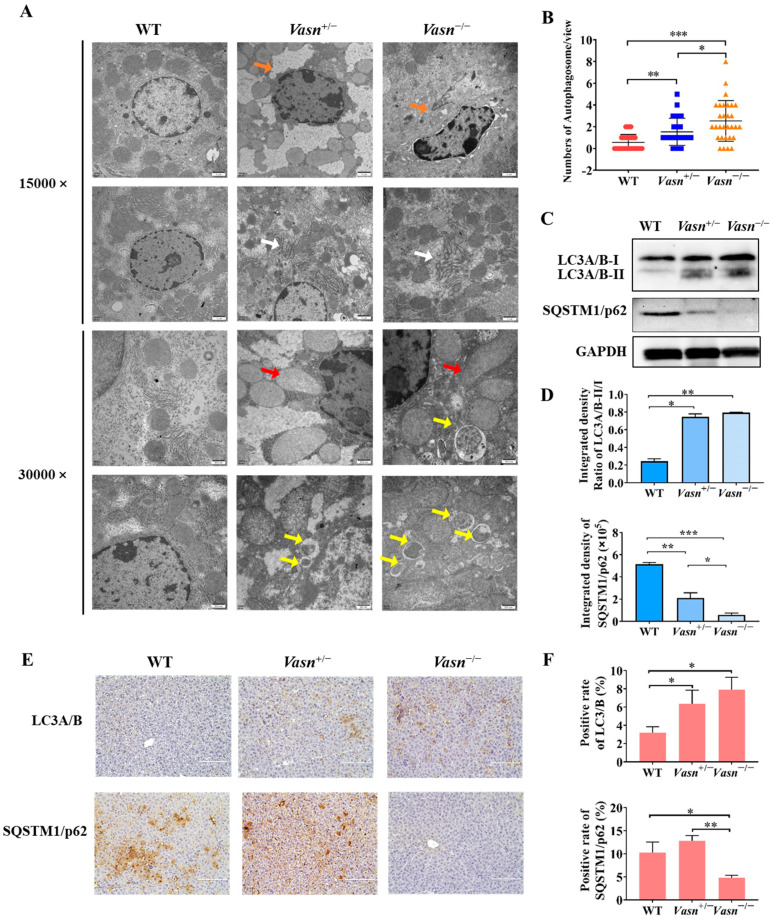
Autophagy induced in *Vasn*^−/−^ and *Vasn*^+/−^ mice. (**A**) TEM observation of hepatocytes of liver tissues from *Vasn*^−/−^, *Vasn*^+/−^, and WT mice at 3 weeks of age; orange arrows show nuclear shrinkage and deformation; white arrows show abnormal endoplasmic reticulum structure; red arrows show mitochondrion swelling; yellow arrows show autophagosomes. (**B**) Quantitative analysis of the number of autophagosomes in each electron microscope photograph (8000×). The statistical analysis data were from 30 photographs per group. (**C**) Upregulation in the protein expression of LC3A/B-II/I and SQSTM1/p62 downregulated in *Vasn*^+/−^ and *Vasn*^−/−^ by Western blot analysis. (**D**) Gray value analysis results of Western blot. (**E**) Immunohistochemical staining analysis of LC3A/B and SQSTM1/p62 expression in the liver of WT mice, heterozygote mice *Vasn*^+/−^, and homozygous mice *Vasn*^−/−^; bar = 100 μm. (**F**) Positive staining of specific antibodies (LC3A/B, SQSTM1/p62) in the liver of WT mice, heterozygote mice *Vasn*^+/−^, and homozygous mice *Vasn*^−/−^; * *p* < 0.05, ** *p* < 0.01, *** *p* < 0.001.

Increasing evidence indicates that autophagy plays a critical role in liver injury [31] and energy metabolism, particularly glycogen metabolism [22]. Hence, we investigated the presence and severity of autophagy in the livers of *Vasn* KO mice to understand the possible cause of liver injury and glycogen reduction triggered by *Vasn* deletion. Ultrastructural observation by TEM revealed minor nuclear shrinkage deformity, chromatin condensation, mitochondrial swelling and vacuolization, and endoplasmic reticulum disorder in hepatocytes of *Vasn*^+/−^ mice. A more significant abnormality in the morphology of organelles in the hepatocytes was observed from the livers of *Vasn*^−/−^ mice, including nuclear shrinkage and deformity, chromatin condensation, nuclear membrane folding, mitochondrial swelling and vacuolization, fuzzy and disordered endoplasmic reticulum, and an increase in the number of lysosomes. In the hepatocytes of the WT mice (used as a control), the chromatin was evenly distributed, without pyknosis and margination, and the endoplasmic reticulum and mitochondria cristae were clear, without edema and vacuole-like changes in the hepatocytes of WT (Figure 4A). Unexpectedly, an increased number of double-membrane-structured autophagosomes were formed in the hepatocytes of *Vasn*^+/−^ and *Vasn*^−/−^ mice (Figure 4B). This finding strongly supported that *Vasn* deficiency induced hepatocyte autophagy in mice.

Autophagic flux was analyzed by measuring the expression of microtubule-associated protein light chain 3 (LC3) and SQSTM1/p62 [32]. LC3 is the most widely used autophagosome marker. The amount of LC3-II correlates with the extent of autophagosome formation [33]. P62 is an autophagic substrate that is engulfed by autophagosomes and degraded after fusion. Therefore, we performed Western blot analysis, and the results revealed a significant increase in the expression levels of LC3A/B-II/I in the livers of *Vasn*^+/−^ and *Vasn*^−/−^ mice compared to WT, while the expression of the autophagic substrate SQSTM1/p62 decreased (Figure 4C,D). These results were independently corroborated by immunohistochemistry analysis (Figure 4E,F). Taken together, these results confirm that *Vasn* deficiency induces hepatocyte autophagy.

### 3.5. Hepatic Differential Expressed microRNA Profile Shows That Vasn Deficiency Regulates the mTOR-ULK1 Pathway

**Figure 5:**
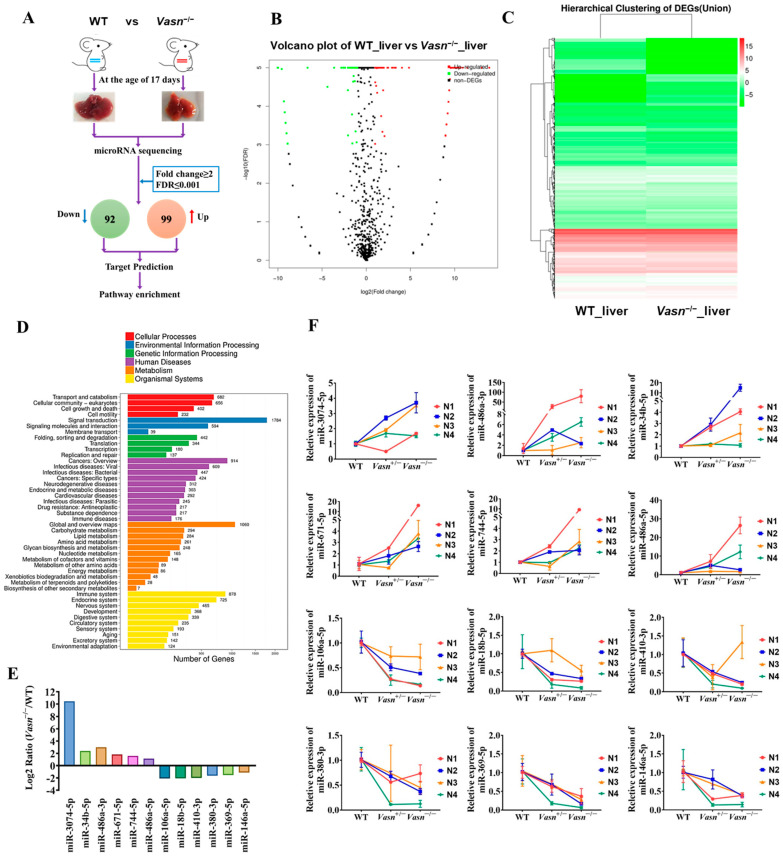
miRNA sequencing (miRNA-seq) analysis provided insight into the differences between the livers of *Vasn* KO homozygous mice and WT mice. (**A**) Schematic of miRNA-seq analysis performed on the livers of *Vasn* KO homozygous mice and WT mice. (**B**) Volcano plot showing differentially expressed miRNAs between the livers of *Vasn*^−/−^ and WT mice. (**C**) Hierarchical clustering of gene expression between liver of *Vasn* KO homozygous *Vasn*^−/−^ mice and WT mice; red: high expression, blue: low expression. (**D**) KEGG enrichment analysis. (**E**) miRNA sequencing results of miR-3074-5p, miR-486a-3p, miR-34b-5p, miR-671-5p, miR-744-5p, miR-486a-5p, miR-106a-5p, miR-18b-5p, miR-410-3p, miR-380-3p, miR-369-5p, and miR-146a-5p in the liver of WT mice and homozygous mice *Vasn*^−/−^. (**F**) qRT-PCR analysis of the expression of miR-3074-5p, miR-486a-3p, miR-34b-5p, miR-671-5p, miR-744-5p, miR-486a-5p, miR-106a-5p, miR-18b-5p, miR-410-3p, miR-380-3p, miR-369-5p, and miR-146a-5p in the liver of WT mice, heterozygote mice *Vasn*^+/−^, and homozygous mice *Vasn*^−/−^. Twelve mice from WT, *Vasn*^+/−^, and *Vasn*^−^^/^^−^ groups were then allocated to different blocks (N1, N2, N3, and N4) using a randomized block design.

Upon confirming the increase in cellular autophagy, the open question becomes the specific mechanism by which *Vasn* deletion activates hepatocyte autophagy mediated by glycogen regulation. MicroRNAs (miRNAs) are considered the main players in regulating autophagy in multiple diseases [34], and some miRNAs from the exosomes are indicative of the mechanism of liver injury disease [35]. The analysis of the alteration pattern of miRNA expression will help clarify how *Vasn* deletion causes hepatocyte autophagy and thereby the induction of liver injury and glycogen decline. Therefore, we applied miRNA sequencing (miRNA-seq) to analyze the profile of the differentially expressed miRNAs (DE-miRNAs). A total of 191 DE-miRNAs were revealed in the transcriptome comparison (Table S2), 99 of which were upregulated and 92 were downregulated in livers of *Vasn*^−/−^ mice (Figure 5A–C). There is a clear difference in KEGG analysis between WT and *Vasn*^−/−^ mice in terms of miRNA expression (Figure 5D).

To validate the miRNA sequencing data, qRT-PCR was performed to analyze the relative expression of thirteen selected miRNAs, including six upregulated miRNAs (miR-3074-5p, miR-34b-5p, miR-486a-3p, miR-671-5p, miR-744-5p, and miR-486a-5p) and seven downregulated miRNAs (miR-532-3p, miR-106a-5p, miR-18b-3p, miR-410-3p, miR-380-3p, miR-369-5p, and miR-146a-5p). The result was consistent with the transcriptome analysis (Figure 5E,F), indicating that the miRNA sequencing data were of good quality and reliable, which was important for further analysis.

**Figure 6:**
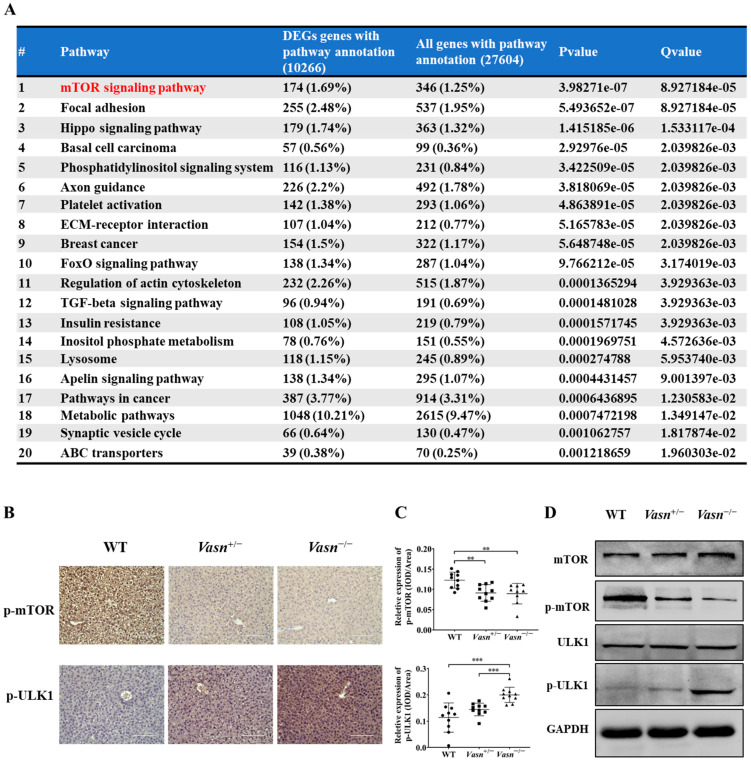
*Vasn* deletion induced autophagy by regulating the mTOR-ULK1 signaling pathway. (**A**) Pathway enrichment results of target genes of differential expression microRNAs, where the mTOR pathway is of the Top 1 that has been significantly enriched (red highlightened). (**B**) Immunohistochemical analysis of mTOR signaling protein expression in the liver of WT, *Vasn*^+/−^, and *Vasn*^−/−^ mice; bar = 100 μm. (**C**) Quantitative integrated optical density (IOD) analysis of immunohistochemical staining from ten mice per group, and three photos per sample. ** *p* < 0.01, *** *p* < 0.001. (**D**) *Vasn* deletion significantly decreased the expression of phosphorylated mTOR (S2448) and increased expression of the phosphorylated ULK1 (S555), confirmed by Western blot analysis.

**Figure 7:**
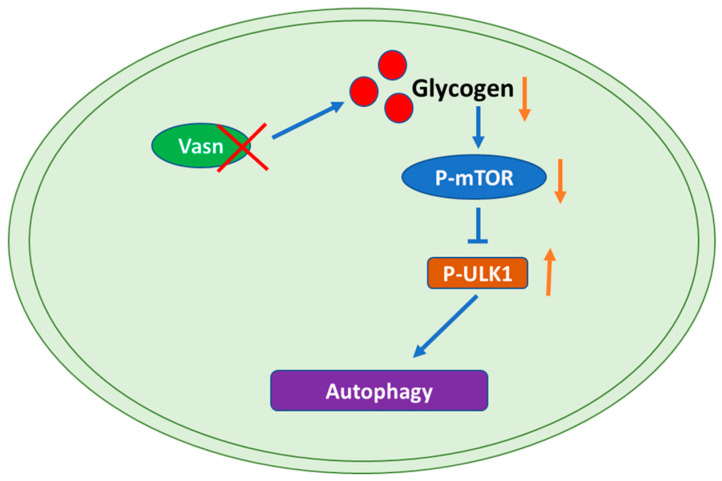
Graphical representation of the molecular mechanism of the role of *Vasn* in autophagy by regulating the mTOR-ULK1 signaling pathway. *Vasn* deficiency induces autophagy by inhibiting glycogen-mediated mTOR phosphorylation and activates the ULK1 signaling pathway.

To identify any potential signaling pathways of *Vasn* upon autophagy, KEGG pathway analysis showed that DE-miRNA target genes had an enrichment for pathways related to the mammalian target of rapamycin (mTOR) signaling pathway (Figure 6A and Table S3). The glucagon signaling pathway related to glycogen metabolism and the autophagy animal pathway were ranked 61st and 62nd, respectively (Tables S4 and S5). mTOR is core hub for the regulatory network of autophagy in response to nutritional status and is regulated by a variety of upstream signaling pathways [21,36]. Under starvation conditions (glucose deprivation), mTOR is inactivated and ULK1 kinase activity increases (e.g., phosphorylation of ULK1 S555) [37]. Thus, we assessed the phosphorylation levels of mTOR (S2448) and ULK1 (S555). Immunohistochemistry indicated that the phosphorylation of mTOR (S2448) was significantly lower in the *Vasn*^+/−^ and *Vasn*^−/−^ groups than in the WT group. The trend of phosphorylation of ULK1 (S555) differed from that of mTOR phosphorylation (Figure 6B,C). Western blot further confirmed that *Vasn* deletion led to significantly decreased phosphorylated mTOR (S2448) protein levels, and increased the phosphorylation of ULK1 (S555), while there was no statistically significant difference in the levels of mTOR and ULK1 between WT and *Vasn* KO mice (Figure 6D). The mTOR signaling pathway globally regulates metabolic signals, organismal growth, and homeostasis in cells by a variety of fundamental signals [38]. mTORC1 signaling reportedly plays an important role in glucose–glycogen homeostasis [39]. Altogether, these data support the hypothesis that *Vasn* deficiency could lead to a decrease in hepatic glycogen, which induces hepatocyte autophagy by inhibiting the mTOR signaling pathway and activating the ULK1 phosphorylation pathway (summarized in Figure 7).

## 4. Discussion

In this study, starting from the initial discovery of weight loss and life span shortening as the unexpecting biological problems of *Vasn* KO mice, we found the association of liver injury and liver glycogen depletion at the macroscopic histology level, followed by the potential involvement of mTOR pathway-mediated hepatocyte autophagy in glucose metabolism disorder of the liver at the microscopic cellular biology and molecular mechanism levels. To our best knowledge, this is the first report to discuss the role of *Vasn* in liver metabolism and autophagy regulation, constituting a reference for related scientific research on *Vasn*.

The vital role of *Vasn* in the progression of several diseases at the cellular level has been well documented [1,5,6,7,8,9,10]. *Vasn* promotes proliferation in prostate cancer [40] and laryngeal cancer [41]. Various studies have demonstrated the role of *Vasn* in the pathogenesis of liver diseases. For instance, *Vasn* promotes cell proliferation and migration, and further inhibits apoptosis in hepatocellular carcinoma (HCC) [6]. Mitochondria-localized *Vasn* protects cells from TNFα-induced apoptosis, and the partial deletion of the *Vasn* coding sequence leads to increased sensitivity of hepatocytes and TNFα-induced apoptosis [3]. However, the impact and the underlying mechanism of *Vasn* in hepatocyte autophagy in mice have not been investigated. In our studies, we initially found abnormal liver functions and liver injury triggered by *Vasn* knockout in C57BL/6J mice generated by CRISPR/Cas9, according to the obvious hepatic histology and the elevated levels of ALP, ALT, TBIL, DBIL, CHE, and GGT of all *Vasn*^−/−^ mice compared to the WT mice; these alterations were also partially present in *Vasn*^+/−^ mice. It is likely that the deletion of *Vasn* might be responsible for the liver problem and thereby the poor longevity and health status of mice. Over-expression of *Vasn* has been found in multiple solid tumor types and plays a role in the stimulation of tumor progression and angiogenesis. Gene knockdown or overexpression of *Vasn* in cells demonstrated opposite effects on cell migration and tubulogenesis, either inhibition or promotion, and thus affected angiogenesis in solid tumors [6,9,42,43]. From the observations of this study, *Vasn* may also have a similar effect on blood vessel formation in the normal liver, as evidenced by the lighter or pale color of livers in mice with *Vasn* deletion. On the other hand, the unexpected discovery of congenital liver injury and hepatocyte dystrophy in *Vasn* KO mice also provides a valuable theoretical basis to use *Vasn* KO laboratory animals as models for studying liver diseases, especially liver cancers, in the future [6].

Autophagy is a catabolic process that in most cases is critical for the prevention of and recovery from alcohol-induced liver injury since cellular proteins and subcellular organelles such as mitochondria are degraded [44]. However, in a few cases, autophagy promotes chronic ethanol-induced liver injury and steatosis in mice [45]. Several varying mechanisms have been proposed to cause liver injury, including heat stroke, ER stress, hepatocyte apoptosis, and autophagy [46]. Furthermore, excessive autophagy can lead to autophagic cell death of hepatocytes [13]. As a typical ultrastructural feature of autophagy, autophagosomes appear when autophagy occurs in cells, and are a kind of organelle with a double-membrane structure. Upon induction, the isolation membrane or phagophore elongates and subsequently encloses a portion of the cytoplasm, which results in the formation of autophagosome [47]. In our study, the light microscope and TEM results revealed an increase in the number of autophagosomes in the hepatocytes in the liver of both *Vasn*^+/−^ and *Vasn*^−/−^ mice compared to the WT. Moreover, upregulation of LC3A/B-II and downregulation of SQSTM1/p62 in the liver tissue of *Vasn*^+/−^ and *Vasn*^−/−^ were also detected, which further supports the involvement of autophagy in *Vasn* KO mice [48]. Additionally, based on the liver dysfunction and liver injury that we observed in *Vasn* KO mice, we hypothesized that *Vasn* deficiency-induced hepatocyte autophagy may be the culprit in the poor longevity and health status of mice.

Autophagy regulates liver physiology by promoting the degradation of macromolecules and organelles to support the overall energy balance and the metabolism and regeneration of organelles. The cellular process contributes to the development and progression of various liver diseases, including hepatitis, steatosis, fibrosis, cirrhosis, and HCC [49]. Mounting evidence indicates a few critical genes related to liver diseases, which are confirmed to also be involved in the autophagy of hepatocytes [50,51]. Accumulating evidence has revealed that miRNAs, combined with their target genes, regulate cell signaling pathways associated with autophagy [34,52,53]. In pursuit of direct scientific proof of the connection between *Vasn* deficiency and abnormal liver function, we were thus inspired to explore the key hepatic miRNAs and their associated signaling pathways mediated by *Vasn* upon autophagy. Interestingly, our miRNA sequencing results indicated that the DE-miRNAs targeting the mTOR pathway might play a critical role in activating hepatocyte autophagy in *Vasn* KO mice. Mechanistically, we demonstrated that *Vasn* deficiency induces autophagy by inhibiting mTOR and activating the ULK1 signaling pathway. mTORC1 is a key regulator of catabolic processes, especially by controlling autophagy [36]. The cross-talk between mTOR-mediated upstream pathways of autophagy is highly complex. In nutrient-rich conditions, mTORC1 inhibits the initiation of autophagy by inhibiting ULK complexes [54]. Cells respond to starvation by shutting down protein synthesis and by activating catabolic processes, including autophagy, to recycle nutrients [55]. Liver glycogen reserves are important for body glucose metabolism, and their replenishment is hormonally linked to nutritional status [56]. Thus, the severe body weight loss observed in *Vasn* KO mice indicates an overall decline in nutrition, which could be a consequence of the decrease in liver glycogen accumulation, wherein *Vasn* plays a role in the autophagy regulation process by modulating the glycogen metabolism.

Abnormal gene expression is associated with various stress signals and alters biological processes. Recent studies have demonstrated key roles for reactive oxygen species stress in *Vasn*-mediated apoptosis. *Vasn* is a critical factor and bridge between hypoxia and Notch signaling in glioma stem-like cells (GSCs) [8]. *Vasn* protects cells against TNFα- and hypoxia-induced apoptosis [3]. Cobalt chloride-induced hypoxia stimulated a robust elevation of *Vasn* expression, and *Vasn* suppressed TNF-α-induced cell death in human trabecular meshwork (HTM) cells [57]. In this study, for the first time, we report that the deletion of *Vasn* leads to a significant decrease in glycogen levels and autophagy activation. Significant depletion in liver glycogen may indicate that the *Vasn* KO mice suffered starvation and nutritional deficiency. Such changes in glycogen reserve usually occur only after fasting in normal mice; however, the *Vasn* KO mice were breastfed, like the WT mice, until the above conditions developed. Apparently, food factors were not the cause of malnutrition, and an alteration in liver glycogen content in the *Vasn* KO mice was also observed in our study [58]. Various studies report that starvation causes autophagy of the hepatocytes [59,60], and our study revealed the close connection of cell metabolism with autophagy. Autophagy participates in various physiological processes, including adaptation to starvation. Glycogen, insulin, and amino acids are effective inhibitors of hepatic autophagy, and mTOR is one of the major hubs of glucose-sensing pathways [61]. Under nutrient sufficiency, high mTOR activity prevents ULK1 activation [54]. Along with transcription, protein phosphorylation also determines the activity and function of a protein, especially in autophagy and the glycogen metabolism pathway [62,63]. The regulation of these two pathway is more likely to inhibit the protein activities of related key proteins (here, mTOR) by reducing the phosphorylation levels without changing their own expression, which we confirmed by Western blot assay, as shown in Figure 6. In light of this analysis, an in-depth study of the correlation between *Vasn* and the mTOR-ULK1 signaling pathway is still required. Conclusively, our current study highlights the critical role of *Vasn* in the homeostasis of the glycogen metabolism regulatory network in liver physiology and functions, particularly the glycogen–autophagy regulation axis. Future studies are needed to dissect the molecular mechanisms underlying the abnormal glycogen metabolism in *Vasn* KO mice, with the aim to fully solve the mystery of the reduced poor nutrition and shortened life span of mice caused by *Vasn* deficiency, which is valuable for downstream research and transgenic productions.

Other than causing liver abnormality, *Vasn* deficiency in C57BL/6J mice also induced abnormalities in myocardial structure and myocardial disorders, which are important pathological features of cardiac hypertrophy (as shown in our previous study) [14], and both can be key causes responsible for the weight loss and the premature death of *Vasn* KO mice, which are complicated and ambiguous. In addition to its role in autophagy regulation, the anti-apoptotic effect of *Vasn* has been also attracting increasing concern [2,57,64]. Mitochondria-localized *Vasn* protects cells from apoptosis, and the deletion of *Vasn* increases the sensitivity of hepatocytes to TNFα-induced apoptosis [3,5]. Knockdown of *Vasn* in human liver cancer cell line HepG2 resulted in decreased cell proliferation and increased apoptosis [6]. Taking these factors into consideration, as *Vasn* is localized in cellular mitochondria and widely expressed in major organs and various cell types (especially during embryonic development), we propose a bold and new hypothesis: If *Vasn* plays a role in cell development and death by regulating proliferation, apoptosis (type I program death), and autophagy (type II program death) to decide the fate of a greater variety of cell types, probably including stem cells, will deleting *Vasn* interfere with the normal development of multiple organs (not just the liver and the heart, as we reported) starting in the embryonic stage, thus leading to dysplasia, malnutrition, and then early death? Demonstrating this hypothesis will contribute to a comprehensive understanding of the specific roles and mechanisms played by *Vasn* in either normal physiology or cancer pathology. This is also a key question that must be answered before the development and application of *Vasn* gene-editing mice as a disease research model.

## 5. Conclusions

The deletion of *Vasn* in mice in this study induced hepatocyte autophagy and thereby cellular damage, thus causing liver injury and malnutrition in general. This may be attributed to the reduction in glycogen levels, which negatively regulate the mTOR signaling pathway at the molecular level. Our data indicate that the related pathway with *Vasn* as the core could be potentially related to the normal physiological function of liver. The direct connection between *Vasn* and liver injury is enlightening and helpful for a more comprehensive understanding of the potential action of *Vasn* in normal liver physiology and pathogenesis of liver cancers. This study lays an important foundation and also provides basic theory for future research on the physiology and pathology of liver disease-related studies using *Vasn* KO experimental animals as models.

## Data Availability

Not applicable.

## Supplementary Materials

The following supporting information can be downloaded at: https://www.mdpi.com/article/10.3390/nu14173600/s1, Figure S1: CRISPR/Cas9-mediated *Vasn* knockout mice; *Vasn*^−/−^ and *Vasn*^+/−^ were generated. (A) Generation strategy of Vasn knockout mice, DNA sequencing, and BLAST analysis revealed a 2384 bp DNA fragment containing the sequence of *Vasn* that had been knocked out from the genome of C57BL/6 *Vasn*^−/−^ mice. (B) Genotypes of knockout mice were detected by agarose gel electrophoresis. In wild-type mice, a 4023 bp fragment was amplified by PCR with primers P1 and P2, and a 542 bp fragment was amplified by PCR with primers P3 and P4; in heterozygote mice *Vasn*^+/−^, 4023 bp and 1639 bp fragments were amplified by PCR with primers P1 and P2, and a 542 bp fragment was amplified by PCR with primers P3 and P4; in homozygote mice *Vasn*^−/−^, a 1639 bp fragment was amplified by PCR with primers P1 and P2, and the product could not be amplified with primers P3 and P4. Table S1: Primers used in this study. Table S2: Differentially expressed microRNAs were screened between wild-type and *Vasn*^−/−^ mice. Table S3: Bioinformatics predicted the targets of DE-miRNAs. Table S4: Pathway enrichment analysis of DE-miRNA targets between wild-type and *Vasn*^−/−^ mice. Table S5: KEGG enrichment results revealed DE-miRNA targets and related pathways between wild-type and *Vasn*^−/−^ mice.
