# Supplementary material for: Vasorin Deletion in C57BL/6J Mice Induces Hepatocyte Autophagy through Glycogen-Mediated mTOR Regulation
Source: Nutrients. 2022 Aug 31;14(17):3600. doi: 10.3390/nu14173600 (PMC9460126; doi:10.3390/nu14173600)
Supplement: Supplementary file 1 [file nutrients-14-03600-s001.zip › Table S1.pdf]

Table S1 Primers used in this study

Primer name	Nucleotide sequence (5′→3′)	Purpose
P1	ACTTCTGGGGGACAATGCTG	Genotype identification for mice
P2	ACTGGGTTTCTTGCCTTGGT	Genotype identification for mice
P3	GCCTCAACAGTCAGCTTCTC	Genotype identification for mice
P4	CTGATGGGCAACCCTGTACT	Genotype identification for mice
miR-486a-3p-F	CGGGGCAGCTCAGTACAGGAT	qRT-PCR
miR-486a-5p-F	TCCTGTACTGAGCTGCCCCGAG	qRT-PCR
miR-106a-5p-F	CGCAAAGTGCTAACAGTGCAGGTAG	qRT-PCR
miR-18b-5p-F	GCGTAAGGTGCATCTAGTGCTGTTAG	qRT-PCR
miR-410-3p-F	GCGCGAATATAACACAGATGGCCTGT	qRT-PCR
miR-380-3p-F	GCGCGTATGTAGTATGGTCCACATCTT	qRT-PCR
miR-369-5p-F	GCGAGATCGACCGTGTTATATTCGC	qRT-PCR
miR-146a-5p-F	CGCGTGAGAACTGAATTCCATGGGTT	qRT-PCR
miR-3074-5p-F	GTTCCTGCTGAACTGAGCCAG	qRT-PCR
miR-34b-5p-F	TAGGCAGTGTCATTAGCTGATTG	qRT-PCR
miR-671-5p-F	AGGAAGCCCTGGAGGGGCTGGAG	qRT-PCR
miR-744-5p-F	TGCGGGGCTAGGGCTAACAGCA	qRT-PCR
