# Supplementary figures and images for: Vasorin Deletion in C57BL/6J Mice Induces Hepatocyte Autophagy through Glycogen-Mediated mTOR Regulation
Source: Nutrients. 2022 Aug 31;14(17):3600. doi: 10.3390/nu14173600 (PMC9460126; doi:10.3390/nu14173600)

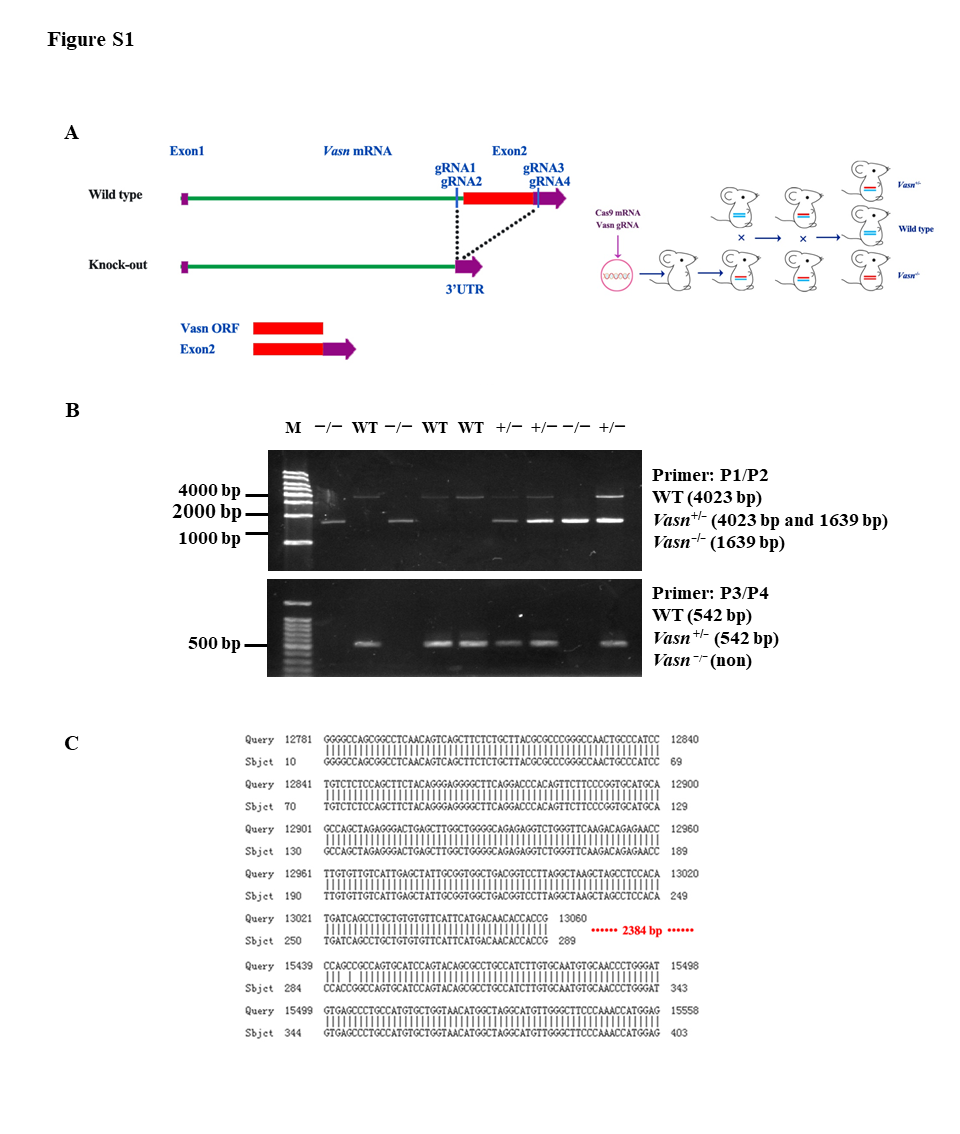

Supplement: Supplementary file 1 [file nutrients-14-03600-s001.zip › Figure S1.tif]
